# Positive effect of dapagliflozin on left ventricular longitudinal function for type 2 diabetic mellitus patients with chronic heart failure

**DOI:** 10.1186/s12933-019-0985-z

**Published:** 2020-01-07

**Authors:** Hidekazu Tanaka, Fumitaka Soga, Kazuhiro Tatsumi, Yasuhide Mochizuki, Hiroyuki Sano, Hiromi Toki, Kensuke Matsumoto, Junya Shite, Hideyuki Takaoka, Tomofumi Doi, Ken-ichi Hirata

**Affiliations:** 1 Division of Cardiovascular Medicine, Department of Internal Medicine, Kobe University Graduate School of Medicine, 7-5-2, Kusunoki-cho, Chuo-ku, Kobe, 650-0017 Japan; 2 Tatsumi Clinic, Himeji, Japan; 3 Division of Cardiology, Osaka Saiseikai Nakatsu Hospital, Osaka, Japan; 4 Division of Cardiology, Aijinkai Takatsuki Hospital, Takatsuki, Japan; 5 Division of Cardiology, Kobe Red Cross Hospital, Kobe, Japan

**Keywords:** Sodium glucose cotransporter type 2 inhibitors, Type 2 diabetes mellitus, Left ventricular diastolic function, Heart failure, Global longitudinal strain

## Abstract

**Background:**

The effect of sodium glucose cotransporter type 2 (SGLT2) inhibitor on left ventricular (LV) longitudinal myocardial function in type 2 diabetes mellitus (T2DM) patients with heart failure (HF) has remained unclear.

**Methods:**

We analyzed data from our previous prospective multicenter study, in which we investigated the effect of the SGLT2 inhibitor dapagliflozin on LV diastolic functional parameters of T2DM patients with stable HF at five institutions in Japan. Echocardiography was performed at baseline and 6 months after administration of dapagliflozin. LV diastolic function was defined as the ratio of mitral inflow E to mitral e′ annular velocities (E/e′). LV longitudinal myocardial function was assessed as global longitudinal strain (GLS), which in turn was determined as the averaged peak longitudinal strain from standard LV apical views.

**Results:**

E/e′ significantly decreased from 9.3 to 8.5 cm/s 6 months after administration of dapagliflozin (p = 0.020) as previously described, while GLS showed significant improvement from 15.5 ± 3.5% to 16.9 ± 4.1% (p < 0.01) 6 months after administration of dapagliflozin. Furthermore, improvement of GLS in HF with preserved ejection fraction patients was more significant from 17.0 ± 1.9% to 18.7 ± 2.0% (p < 0.001), compared to that in HF with mid-range ejection fraction and HF with reduced ejection fraction patients from 14.4 ± 2.4% to 15.5 ± 1.8% (p = 0.06) and from 8.1 ± 1.5% to 7.8 ± 2.1% (p = 0.44), respectively. It was noteworthy that multiple regression analysis showed that the change in GLS after administration of dapagliflozin was the only independent determinant parameters for the change in E/e′ after administration of dapagliflozin.

**Conclusion:**

Dapagliflozin was found to be associated with improvement of LV longitudinal myocardial function, which led to further improvement of LV diastolic function of T2DM patients with stable HF. GLS-guided management may thus lead to improved management of T2DM patients with stable HF.

## Background

Type 2 diabetes mellitus (T2DM) has come to be considered an independent predictor of mortality, and also a contributor to the development of heart failure (HF) in patients with reduced ejection fraction (HFrEF) and with preserved ejection fraction (HFpEF), as well as cardiovascular disease [1, 2]. Diabetes-related cardiomyopathy is currently viewed as a diastolic dysfunction, but was previously considered to be the earliest functional alteration in the course of diabetes-related cardiomyopathy. LV longitudinal myocardial dysfunction, on the other hand, as assessed in terms of lower global longitudinal strain (GLS), has been identified even in T2DM patients with preserved LV ejection fraction (LVEF) but without overt coronary artery disease or HF [3–10], and should thus be considered the first marker of a preclinical form of DM-related cardiac dysfunction, leading to HFpEF. Sodium glucose cotransporter type 2 (SGLT2) inhibitors represent a new class of anti-hyperglycemic agents for T2DM, which act insulin-independently to selectively inhibit renal glucose reabsorption, thereby increasing urinary glucose excretion. A large clinical trial using dapagliflozin, a SGLT2 inhibitor, found that treatment with dapagliflozin of T2DM patients who had or were at risk for atherosclerotic cardiovascular disease, did not result in a higher or lower rate of cardiovascular events than placebo, but did result in a lower rate of cardiovascular death or hospitalization for HF [11]. In addition, a recent other large clinical trial using dapagliflozin demonstrated that dapagliflozin reduced risk of worsening HF or death from cardiovascular causes for patients with HFrEF compared to those who received a placebo, regardless of the presence or absence of T2DM [12]. On the other hand, the effect of SGLT2 inhibitors on LV longitudinal myocardial function in T2DM patients with HF remains uncertain. To examine this effect, as well as the association of LV longitudinal myocardial function with LV diastolic function after administration of SGLT2 inhibitor in T2DM patients with stable HF, we analyzed data from a previous prospective multicenter study of ours, in which we investigated the effect of SGLT2 inhibitor on LV diastolic functional parameters including E/e′ and left atrial volume index (LAVI) of T2DM patients with stable HF at five institutions in Japan [13].

## Methods

### Study population

The details of our prospective multicenter study have been described previously [13]. Briefly, eligible for inclusion in this study were 53 T2DM patients with stable HF from the participating centers, who had been taking at least one antidiabetic drug other than SGLT2 inhibitors for more than 1 year between December 2015 and March 2016. All patients had a previous history of HF, but they were in clinically stable condition at the time of enrollment, defined as an absence of exacerbation of HF symptoms for at least 6 months. Patients were excluded from enrolment study if they met any of the following criteria: 1. age < 20 and > 75 years; 2. type I DM; 3. T2DM with HbA1c < 6.5% and > 10.0%; 4. insulin-dependent T2DM; 5. serious renal dysfunction defined as glomerular filtration rate < 45 mL/min/1.73 m^2^; 6. hypotension < 90/50 mmHg; 7. malignancy; 8. poor nutritional status; and 9. atrial fibrillation. According to the current guideline [14], patients were subsequently categorized as HFrEF, HFpEF or HFmrEF if their LVEF was < 40%, ≥ 50% or 40–49%, respectively. The concentration of biochemical analyses was measured by routine method. Specifically, HbA1c was measured using ADAMS A1c HA-8182 (ARKRAY, Kyoto, Japan) or HLC723G11 or HLC-723G8 (Tosoh, Tokyo, Japan), and BNP was measured using AIA-CL2400 or AIA-900 (Tosoh, Tokyo, Japan) or LUMIPULSE L2400 (FUJIREBIO, Tokyo, Japan). This study was approved by the local ethics committee of our institution (No. B190231).

### Study protocol

Stable HF patients who had been taking at least one antidiabetic drug other than SGLT2 inhibitors and who had consented to their participation in this study, started the administration of dapagliflozin at 5 mg/day. Other drugs were not changed after the start of administration of dapagliflozin. The physical examinations and blood tests were performed at baseline, 3 months, and 6 months after administration of dapagliflozin, while echocardiography was performed at baseline and 6 months after administration of the SGLT2 inhibitor. Only if a patient’s HbA1c had failed to improve by 3 months after administration of dapagliflozin, the dose was raised from 5 to 10 mg/day.

### Standard echocardiographic examination

All patients underwent a resting standard echocardiographic examination using commercially available echocardiography systems (Aplio Artida, Aplio 400 and Xario; Canon Medical Systems, Tochigi, Japan, Vivid E9; GE-Vingmed, Horten, Norway, and iE33 and EPIQ7; Philips Medical Systems, Andover, MA). Digital routine grayscale two-dimensional cine loops from three consecutive heart beats were obtained at end-expiratory apnea from standard parasternal and apical views. Sector width was optimized to allow for complete myocardial visualization while maximizing the frame rate. Standard echocardiographic measurements were obtained in accordance with the current guidelines of the American Society of Echocardiography/European Association of Cardiovascular Imaging [15].

### Speckle-tracking strain analysis for GLS

**Fig. 1:**
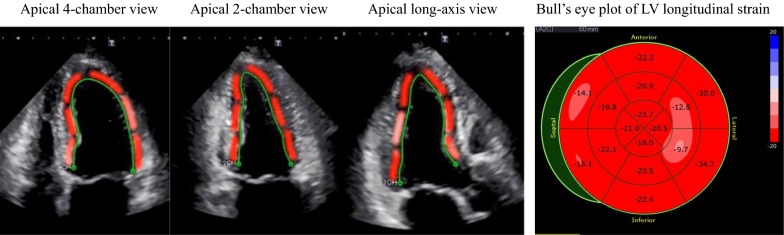
Example of assessment of LV longitudinal myocardial function, expressed as GLS by means of two-dimensional speckle-tracking imaging, showing color-coded speckle-tracking images and the corresponding bull’s eye plot of LV longitudinal strain

Speckle-tracking strain analysis was performed for each patient with the aid of a single dedicated software to evaluate LV longitudinal function, which was assessed in terms of GLS (AutoSTRAIN, TOMTEC-ARENA, TOMTEC Imaging Systems GmbH, Unterschleissheim, Germany). Briefly, apical 4-, 2- and long-axis views with the Digital Imaging and Communications in Medicine (DICOM) formatted file images were uploaded onto a personal computer for subsequent off-line GLS analysis (Fig. 1). Longitudinal speckle-tracking strain was calculated applying an automated contouring detection algorithm, and manual adjustments of regions of interest were performed where necessary. Longitudinal strain results were visualized color-coded in the individual clips and combined in a bull’s eye plot. GLS was then determined as the averaged peak longitudinal strain of 16 LV segments, and was expressed as an absolute value in accordance with current guidelines [15].

### Statistical analysis

Continuous variables were expressed as mean values and standard deviation for normally distributed data, and as the median and interquartile range for non-normally distributed data. Categorical variables were expressed as frequencies and percentages. Paired t tests or Wilcoxon signed-rank test were used for group comparison between baseline and 6 months after the start of administration of dapagliflozin. Independent associations of changes in E/e′ between baseline and 6 months after the start of administration of dapagliflozin with clinical and echocardiographic parameters were evaluated by means of multiple regression analysis. The intraclass correlation coefficient was used to determine inter- and intra-observer reproducibilities for GLS from 20 randomly selected patients using an identical cine-loop for each view. For all steps, p < 0.05 was considered statistically significant. All analyses were performed with commercially available software (SPSS software version 24.0, SPSS Inc., Chicago, IL).

## Results

### Patient characteristics

The baseline clinical and echocardiographic characteristics of the 53 T2DM patients are summarized in Table 1 [13]. Their mean age was 68 years (60–73), LV ejection fraction (LVEF) was 62.3% (49.3–68.3), and 21 patients (38%) were female. The dapagliflozin dose was raised from 5 to 10 mg/day in one patient.

**Table 1:** Baseline characteristics of patients

Clinical characteristics	
Age, years	68 (60–73)
Gender (female), n (%)	21 (38)
Body mass index, kg/m^2^	25.9 ± 5.1
Systolic blood pressure, mmHg	130 ± 16
BNP, pg/mL	27.9 (9.0–58.2)
eGFR, mL/min/1.73 m^2^	70.6 ± 17.0
HbA1c, %	7.2 ± 0.8
HF classification, n (%)	
HFpEF	37 (69)
HFrEF	7 (13)
HFmrEF	9 (17)
Comorbidities, n (%)	
Hypertension	43 (81)
Dyslipidemia	42 (79)
Cardiovascular event	12 (21)
Medications, n (%)	
CCB	19 (36)
ACEI/ARB	42 (79)
β-Blocker	27 (51)
Diuretics	10 (19)
Statin	37 (70)
Antidiabetic drugs	
DPP-4I	40 (75)
GLP-1 RA	1 (2)
SU	11 (21)
α-GI	9 (17)
Thiazolidinedione	11 (21)
Metformin	14 (26)
Echocardiographic parameters	
LV end-diastolic volume, mL	74.2 (55.1–104.1)
LV end-systolic volume, mL	24.7 (17.0–54.5)
LVEF, %	62.3 (49.3–68.3)
LVMI, g/m^2^	75.0 (61.7–92.0)
LAVI, mL/m^2^	31 (23–45)
E/e′	9.3 (7.7–11.8)

Data are mean ± SD for normally distributed data and median and interquartile range for non-normally distributed data, or n (%)

*DM* diabetes mellitus, *BNP* plasma brain natriuretic peptide, *HF* heart failure with preserved ejection fracti, *HFrEF* heart failure with reduced ejection fracti, *HFm* heart failure with mid-range ejection fraction, *CCB* calcium channel block, *ACEI* angiotensin-converting enzyme inhibit, *ARB* angiotensin II receptor block, *DPP-4I* Dipeptidyl Peptidase-4 inhibit, *GLP-1 RA* glucagon-like peptide-1 receptors agonists, *SU* Sulfonylureas, *α-GI* α-glucosidase inhibitors, *LVEF* left ventricular ejection fraction, *LVMI* left ventricular mass index, *LAVI* left atrial volume index, *E* peak early diastolic mitral flow velocity, *e′* Spectral pulsed-wave Doppler-derived early diastolic velocity from the septal mitral annulus

### Change in GLS at baseline and 6 months after administration of dapagliflozin

**Fig. 2:**
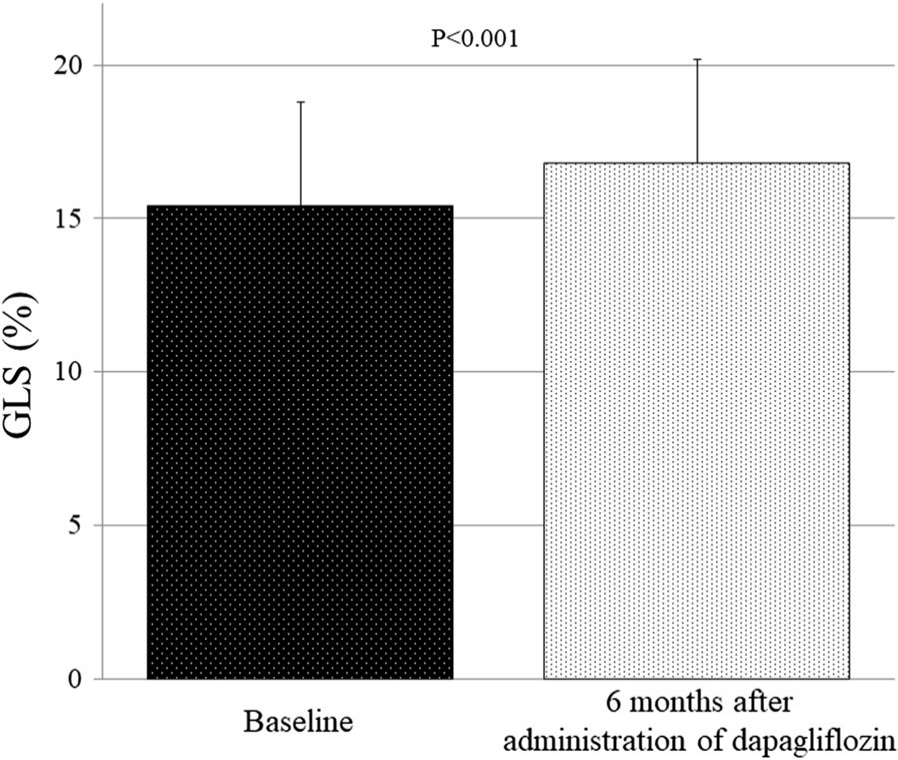
Bar graphs of GLS at baseline and 6 months after administration of dapagliflozin, showing significant improvement of GLS

All clinical and echocardiographic characteristics including LV diastolic function of the 53 T2DM patients at baseline and 6 months after administration of dapagliflozin are summarized in Table 2 [13]. E/e′ significantly decreased from 9.3 to 8.5 cm/s 6 months after administration of dapagliflozin (p = 0.020) as previously described. GLS showed significant improvement from 15.4 ± 3.4% to 16.8 ± 4.0% (p < 0.001) 6 months after administration of dapagliflozin (Fig. 2).

**Table 2:** Comparison of variables between baseline and 6 months after the administration of dapagliflozin

	Baseline	6 months	*p* value
Clinical characteristics			
Body mass index, kg/m^2^	25.9 ± 5.1	25.4 ± 5.1	< 0.001
Systolic blood pressure, mmHg	130 ± 16	128 ± 18	0.218
BNP, pg/mL	27.9 (9.0–58.2)	28.9 (9.6–62.9)	0.132
eGFR, mL/min/1.73 m^2^	70.6 ± 17.0	65.6 ± 15.3	0.001
HbA1c, %	7.2 ± 0.8	7.0 ±0.8	0.108
Echocardiographic parameters			
LV end-diastolic volume, mL	74.2 (55.1–104.1)	68.5 (54.8–93.8)	0.270
LV end-systolic volume, mL	24.7 (17.0–54.5)	20.5 (15.2–57.1)	0.105
LVEF, %	62.3 (49.3–68.3)	63.6 (55.3–71.0)	0.011
LVMI, g/m^2^	75.0 (61.7–92.0)	67.0 (55.0–81.9)	<0.001
LAVI, mL/m^2^	31 (23–45)	26 (21–32)	0.001
E/e′	9.3 (7.7–11.8)	8.5(6.6–10.7)	0.020

Data are mean ± SD for normally distributed data and median and interquartile range for non-normally distributed data, or n (%)

Abbreviation as in Table 1

**Fig. 3:**
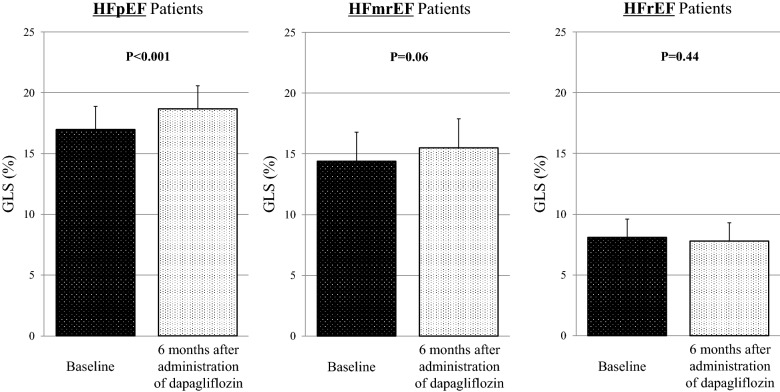
Bar graphs of GLS at baseline and 6 months after administration of dapagliflozin in HFpEF, HFmrEF, and HFrEF patients, showing that improvement of GLS in HFpEF patients was more significant compared to that in HFmrEF and HFrEF patients

Furthermore, improvement of GLS 6 months after administration of dapagliflozin in HFpEF patients was more significant from 17.0 ± 1.9% to 18.7 ± 2.0% (p < 0.001), compared to that in HFmrEF and HFrEF patients from 14.4 ± 2.4% to 15.5 ± 1.8% (p = 0.06) and from 8.1 ± 1.5% to 7.8 ± 2.1% (p = 0.44), respectively (Fig. 3).

### Parameters for change in E/e′ after administration of dapagliflozin

Table 3 shows the results of the multiple regression analysis for the associations of LV diastolic function assessed in terms of E/e′ with clinical and echocardiographic parameters 6 months after administration of dapagliflozin. An important finding of the multiple regression analysis was that a relative change in GLS was the only independent determinant parameter for a relative change in E/e′ 6 months after administration of dapagliflozin.

**Table 3:** Multiple regression analysis for the association of E/e′ after administration of dapagliflozin

Independent variables	Coefficient	Standard error	T value	p value
Body mass index	− 0.06	1.917	− 0.035	0.973
Systolic blood pressure	− 0.710	0.604	− 1.174	0.254
Heart rate	0.321	0.846	0.380	0.708
HbA1c	− 7.748	16.254	− 0.477	0.639
LVEF	0.204	1.107	0.184	0.856
LV end-systolic volume	− 0.132	0.433	− 0.306	0.763
E/e′	− 1.689	0.863	− 1.958	0.06
GLS	2.572	4.720	0.545	0.591
ΔBody mass index	4.097	3.098	1.322	0.201
ΔSystolic blood pressure	0.250	0.393	− 1.328	0.199
ΔHbA1c	− 2.396	1.805	− 1.328	0.199
ΔGLS	− 1.562	0.720	− 2.169	0.041
ΔLVMI	0.334	0.530	0.630	0.536

*ΔHbA1c* a relative change in HbA1c 6 months after administration of dapagliflozin, *ΔGLS* a relative change in GLS 6 months after administration of dapagliflozin, *ΔLVMI* a relative change in LVMI 6 months after administration of dapagliflozin. Other abbreviations as in Table 1

### Reproducibility

The intraclass correlation coefficient for inter-observer reproducibility of GLS was 0.954 (95% confidence interval: 0.888–0.982), and the intraclass correlation coefficient for intra-observer reproducibility of GLS was 0.986 (95% confidence interval: 0.965–0.994).

## Discussion

The findings of our study indicate that LV longitudinal myocardial function, assessed in terms of GLS for T2DM patients with stable HF, had significantly improved 6 months after administration of dapagliflozin. In addition, improvement of GLS for HFpEF patients was superior to that for non-HFpEF patients. Importantly, improvements of GLS were strongly associated with those in E/e′ 6 months after administration of dapagliflozin.

### LV longitudinal myocardial function in T2DM

The presence of T2DM has come to be considered an independent predictor of mortality, and also a contributor to the development of HF in patients with HFrEF and HFpEF [1, 2]. It is particularly well known that T2DM is a major cause of HFpEF, while the presence of T2DM is associated with outcome for patients with HFrEF as well as HFpEF. In fact, Sakakibara et al. reported that the prognosis of HFrEF patients due to non-ischemic dilated cardiomyopathy with T2DM was worse than that of those without T2DM, while multivariate analysis showed that T2DM was significantly associated with an increase in the incidence of cardiac events [16]. Diabetes-related cardiomyopathy is presented as a diastolic dysfunction, which had previously been considered the earliest functional alteration in the course of diabetes-related cardiomyopathy. Moreover, LV diastolic dysfunction is thought to be an underlying pathophysiological abnormality of patients with HFpEF, and thus its assessment plays an important role in diagnosis. In addition, it has been reported that LV diastolic dysfunction is also independently associated with outcomes in patients with HFrEF as well as HFpEF [14, 17].

On the other hand, LV longitudinal myocardial dysfunction has been identified even in T2DM patients with preserved LVEF but without overt coronary artery disease and overt HF [3–10], so that it, rather than LV diastolic dysfunction, should be currently considered the first marker of a preclinical form of diabetes-related cardiac dysfunction, possibly leading to HFpEF. Ernande et al. showed that LV longitudinal myocardial dysfunction detected as GLS < 18% was present even in T2DM patients with preserved LVEF and even those with normal LV diastolic function [18]. In addition, various factors such as acute hyperglycemia [19] and obesity [20, 21] were associated with LV longitudinal myocardial dysfunction in asymptomatic DM patients with preserved LVEF. Our group also previously showed that LV longitudinal myocardial function was strongly and independently of age associated with LV diastolic function in DM patients with preserved LVEF, in contrast to in normal subjects with age-related LV diastolic dysfunction [8]. Thus, it has been suggested that the presence of T2DM leads to LV longitudinal myocardial dysfunction as well as LV diastolic dysfunction, that LV longitudinal myocardial dysfunction is associated with LV diastolic function, and that reduced LV longitudinal myocardial function can coexist with LV diastolic dysfunction in T2DM patients with preserved LVEF, leading to HFpEF. Moreover, improvement of GLS after administration of dapagliflozin for HFpEF patients was superior to that for non-HFpEF patients in this study. Since LV longitudinal myocardial dysfunction can be considered as the early functional alteration in HF patients, positive effect of dapagliflozin on LV longitudinal function was shown in HF patients whose LV dysfunction was milder (preserved LVEF) rather than those with already severe LV dysfunction (reduced LVEF). Thus, these findings may lead to early intervention of SGLT2 inhibitors for HF patients with T2DM.

Leung et al. showed that improvements in glycemic control by antidiabetic drugs including insulin (not including SGLT2 inhibitors) led to improvements in GLS as well as LV diastolic function in asymptomatic T2DM patients with preserved LVEF during 12-month follow-up [22]. Moreover, they showed patients who lowered their HbA1c by ≥ 1.0% had a significantly higher relative improvement in GLS than those who did not. Thus, there is a possibility that improvement in GLS might be resulted from better glycemic control irrespective of SGLT2 inhibitors.

### Impact of SGLT2 inhibitor on cardiovascular events in T2DM

DECLARE-TIMI 58 was a large trial that assessed cardiovascular outcomes for 17,160 patients who were treated with the SGLT2 inhibitor dapagliflozin and followed for a median of 4.2 years [11]. Main findings from the DECLARE-TIMI 58 trial were that T2DM patients who were at high risk of cardiovascular events, dapagliflozin was noninferior to placebo with respect to the composite safety outcome of major adverse cardiovascular events, but neither did it result in a significantly lower rate of major adverse cardiovascular events than placebo. Dapagliflozin did result in a lower rate of the other prespecified primary efficacy outcomes such as the composite of cardiovascular death or hospitalization for HF, which was reflected in a lower rate of hospitalization for HF. Large clinical trials using other SGLT2 inhibitors such as empagliflozin (EMPA-REG OUTCOME trial) [23] and canagliflozin (CANVAS Program) [24] also found that T2DM patients at high risk of cardiovascular events derived cardiovascular benefits from the SGLT2 inhibitor as compared to a placebo. Importantly, a recent large clinical trial, DAPA-HF, which comprised 4744 patients with HFrEF, showed that the risk of the primary composite outcome of worsening HF or death from cardiovascular causes was significantly lower for the dapagliflozin than for the placebo group, regardless of the presence or absence of T2DM [12].

It is well known that SGLT2 inhibitor is associated with lower blood pressure and weight loss as well as a reduction in HbA1c levels, changes which in turn have a significant impact on LV function. Moreover, canagliflozin, an SGLT2 inhibitor, reduced the risk of kidney failure as well as cardiovascular events compared to the placebo group for T2DM patients with albuminuric chronic kidney disease, followed up for a median of 2.62 years [25]. Therefore, these multifaceted effects of SGLT2 inhibitors on such risk factors of LV diastolic function may well lead to improvement for T2DM patients with LV diastolic function. Our recent prospective multicenter trial using T2DM patients with stable HF showed that use of the SGLT2 inhibitor dapagliflozin was associated with improvements in LV diastolic functional parameters including E/e′ and left atrial volume index. Another group also showed positive effect of SGLT2 inhibitors on LV systolic and diastolic function [26], whereas, Roy et al. recently reported that SGLT-2 inhibitors did not appear to the improvement of LV diastolic functional parameters in HFpEF patients with T2DM [27]. In addition, an interesting large randomized clinical trial known as EMPEROR-Preserved Trial is currently in progress. This trial will investigate the safety and efficacy of empagliflozin in approximately 5750 patients with HFpEF regardless of the presence or absence of T2DM [28]. Thus, SGLT2 inhibitors may open a new window on the treatment of HF by the findings from this trial.

### Clinical implications

Although LV diastolic function also plays an important role for patients with HFrEF as well as HFpEF in the development of cardiovascular events and outcomes, LV longitudinal myocardial function assessed in terms of GLS has been reported to be a sensitive marker of early subtle abnormalities of LV myocardial performance, helpful for the prediction of outcomes for various cardiac diseases, and superior to conventional echocardiographic indices [29–33]. Thus, the utility for HF patient management of GLS in conjunction with HF stage classification rather than of conventional echocardiographic parameters has come to be widely reported [29]. For HF patients, GLS can be useful for the prediction of subclinical LV dysfunction, thus identifying patients more at risk of progressing to HF stage or identifying in detail disease severity or prognosis. Since the presence of T2DM is an independent risk factor for the development of HF and its associated mortality, interest in the assessment of precise risk stratification in T2DM patients has remained strong. In our study reported here, the improvement of LV diastolic function after administration of dapagliflozin was found to be strongly associated with that of in GLS. Thus, effect of SGLT2 inhibitors on LV function is multifactorial [34–36], so that GLS-guided management using dapagliflozin for T2DM with stable HF may therefore potentially be able to prevent progression to later HF and may offer new insights into the management of T2DM with HF.

### Study limitations

This study comprised a small number of patients and did not use a placebo-controlled group, so that future prospective studies with larger patient populations including placebo-controlled groups will be needed to validate our findings. In fact, EMPA-HEART trial of a prospective study is currently in progress [37]. In this trial, T2DM patients with preserved LVEF are randomized to either empagliflozin or sitagliptin to investigate a change in GLS from baseline to 1 and 6 months after treatment initiation. Furthermore, the use of thiazolidinedione is contraindicated in HF patients, but 11 patients (21%) used thiazolidinedione in this study. When we re-analyzed except for these 11 patients, overall results were similar. Finally, the dose of all randomized clinical trial with dapagliflozin including DECLARE-TIMI 58 was 10 mg/day. However, only one patient received 10 mg/day of dapagliflozin, and others received 5 mg/day of dapagliflozin in this study. Since the starting dose of dapagliflozin was 5 mg/day in Japan, this difference may occur.

## Conclusion

Dapagliflozin was shown to be associated with improvement of LV longitudinal myocardial function, leading to further improvement of LV diastolic function for T2DM patients with stable HF. GLS-guided management may thus have potential for better management of T2DM patients with stable HF.

## Data Availability

Data sharing not applicable to this article as no datasets were generated or analyzed during the current study.

## Abbreviations

DICOM: digital imaging and communications in medicine
E: early diastolic trans-mitral flow wave velocity
e′: spectral pulsed-wave Doppler-derived early diastolic velocity from the septal mitral annulus
GLS: global longitudinal strain
HF: heart failure
HFmrEF: heart failure with mid-range ejection fraction
HFpEF: heart failure with preserved ejection fraction
HFrEF: heart failure with reduced ejection fraction
LAVI: left atrial volume index
LV: left ventricular
LVEF: left ventricular ejection fraction
SGLT2: sodium glucose cotransporter type 2
T2DM: type 2 diabetes mellitus
